# Icariin Attenuates OGD/R-Induced Autophagy via Bcl-2-Dependent Cross Talk between Apoptosis and Autophagy in PC12 Cells

**DOI:** 10.1155/2016/4343084

**Published:** 2016-08-16

**Authors:** Zhen-tao Mo, Wen-na Li, Yu-rong Zhai, Qi-hai Gong

**Affiliations:** ^1^Zunyi Medical University, Zhuhai Campus, Zhuhai, Guangdong 519041, China; ^2^Department of Pharmacology and Key Laboratory of Basic Pharmacology of Ministry of Education, Zunyi Medical University, Zunyi 563000, China

## Abstract

Icariin (ICA), an active component of* Epimedium brevicornum* Maxim, exerts a variety of neuroprotective effects such as antiapoptosis. However, the mechanisms underlying antiapoptosis of ICA in neurons exposed to oxygen-glucose deprivation and reperfusion (OGD/R) are unclear. The B-cell lymphoma-2 (Bcl-2) protein family plays an important role in the regulation of apoptosis and autophagy through Bcl-2-dependent cross talk. Bcl-2 suppresses apoptosis by binding to Bax and inhibits autophagy by binding to Beclin-1 which is an autophagy related protein. In the present study, MTT result showed that ICA increased cell viability significantly in OGD/R treated PC12 cells (*P* < 0.01). Results of western blotting analysis showed that ICA increased Bcl-2 expression significantly and decreased expressions of Bax, cleaved Caspase-3, Beclin-1, and LC3-II significantly in OGD/R treated PC12 cells (*P* < 0.01). These results suggest that ICA protects PC12 cells from OGD/R induced autophagy via Bcl-2-dependent cross talk between apoptosis and autophagy.

## 1. Introduction

Oxygen-glucose deprivation and reperfusion (OGD/R) result in neuronal apoptosis and autophagy. Apoptosis is a process of programmed cell death. Autophagy, a pathway of cellular protein degradation, is usually activated by starvation, ischemia, growth factor deficit, and so forth and helps to maintain cellular protein homeostasis [1–3], but excessive activation of autophagy may also result in autophagic cell death and apoptosis [4–6]. The B-cell lymphoma-2 (Bcl-2) family of proteins plays an important role in cross talk between apoptosis and autophagy. The Bcl-2 family of proteins consists of antiapoptotic members such as Bcl-2 and Bcl-xL and proapoptotic members such as Bax and Bak. Bcl-2 suppresses apoptosis by binding to Bax and inhibits autophagy by binding to Beclin-1 which is an autophagy related protein [7–9].

ICA, an active flavonoid of* Epimedium brevicornum* Maxim, possesses various neuroprotective effects including anti-inflammation, antioxidation, and antiapoptosis [10–12]. It was reported that ICA protected neurons against OGD induced apoptosis [13]. However, the underlying mechanisms are incompletely understood. Whether ICA can protect cells from OGD induced autophagy via Bcl-2-dependent cross talk between apoptosis and autophagy is unknown. Therefore, in this study, we evaluated the abovementioned potential mechanisms of ICA in rat pheochromocytoma PC12 cells which were widely used in the study of neuropharmacology [14, 15].

## 2. Materials and Methods

### 2.1. ICA Preparation

ICA (purity, 98.3% by HPLC) was purchased from Nanjing Zelang Pharmaceutical Technology Co., Ltd. (Nanjing, China). ICA was dissolved in dimethyl sulfoxide (DMSO) to 10^-3 ^M and stored at −20°C. When ICA was used, 10^-3 ^M ICA was diluted with Earle's balanced salt solution or full culture medium to the final concentration of 10^-5 ^M. The composition of Earle's balanced salt solution and full culture medium is previously reported [14]. Earle's balanced salt solution was composed of 116 m M NaCl, 5.4 mM KCl, 1 mM NaH_2_PO_4_, 0.9 mM CaCl_2_, 0.8 mM MgSO_4_, and 10 mg/L phenol red. The full culture medium contained 4.5 g/mL glucose, 5% foetal bovine serum (Gibco, USA), and 5% horse serum (Gibco, USA) in Dulbecco's modified Eagle's medium (DMEM).

### 2.2. Cell Cultures

PC 12 cells (American Type Culture Collection, CRL-1721) were cultured as previously described [14]. PC12 cells were cultured in full culture medium and were incubated at 37°C in 5% CO_2_. Culture medium was refreshed every two days.

### 2.3. OGD/R

PC12 cells were deprived of oxygen and glucose for 2 h to simulate ischemic injury in vitro as previously described [14]. PC12 cells were rinsed with phosphate buffer solution (PBS) once and incubated in Earle's balanced salt solution as described above. Then, the cells were incubated in a hypoxia chamber (HF100, Heal Force, China) with 1% O_2_, 94% N_2_, and 5% CO_2_ for 2 h. After deprivation of glucose and hypoxia, Earle's balanced salt solution was removed and the cells were cultured in full culture medium under normoxic conditions for 24 h. Normal control cells were refreshed with full culture medium and cultured in a CO_2_ incubator (HF90, Heal Force, China) under normoxic conditions.

### 2.4. Drug Administration

PC12 cells were pretreated with 10^−5^ M ICA or 10 *μ*M nimodipine in full culture medium under normoxic conditions for 1 h before hypoxia. The cells were washed with PBS once and incubated in Earle's balanced salt solution supplemented with 10^−5^ M ICA or 10 *μ*M nimodipine under hypoxia conditions for 2 h. Based on our pilot experiment, we have used ICA at the concentrations of 10^−7^, 10^−6^, and 10^−5^ M and found the concentration of 10^−5^ M is effective and appropriate.

### 2.5. Metabolic 3-(4,5-dimethylthiazol-2-yl)-2,5-diphenyltetrazolium Bromide (MTT) Assay

0.1 mL of cells (1 × 10^5^ cells/mL) was seeded into 96-well plates. The cells were divided into four groups: normal group, model group, nimodipine group, and ICA group. Each group contained 10 samples. After growth for two days, the cells were treated with OGD/R and drug administration as described above. MTT assay was performed as previously described [14]. Absorbance was measured at 570 nm on an ELISA reader (Multiskan Mk3, Thermo Scientific, USA).

### 2.6. Western Blotting Analysis

It contained 6 samples in normal group, model group, and drug treated groups, respectively. Cell lysates were prepared by incubation in RIPA lysis buffer containing protease inhibitors. Protein concentrations were measured with a BCA kit. Approximately 30 *µ*g of total protein was separated by SDS-PAGE (5%–10%) and then transferred to polyvinylidene fluoride (PVDF) membranes. Membranes were blocked with 5% nonfat dry milk for 60 min at room temperature and incubated with primary antibodies against Bcl-2 (1 : 1000, Cell Signaling Technology, USA), Bax (1 : 1000, Cell Signaling Technology, USA), Beclin-1 (1 : 1000, Cell Signaling Technology, USA), LC3 (1 : 1000, Novus Biologicals, USA), Caspase-3 (1 : 1000, Cell Signaling Technology, USA), and GAPDH antibodies (1 : 1000, Millipore, USA) at 4°C overnight followed by incubation with HRP-conjugated goat anti-rabbit IgG antibody (1 : 5000, Shanghai Kangchen Biotechnology, China) at room temperature for 90 min. The immunoblots were visualized using enhanced chemiluminescence provided with the ECL kit (Millipore, USA) and exposure to film. The density of blots was quantified using Band-Scan software.

### 2.7. Statistical Analysis

Data were expressed as the mean ± SD. Statistical significance was analyzed with one-way ANOVA. *P* value of less than 0.05 was considered significant.

## 3. Results

### 3.1. ICA Increased Cell Viability in OGD/R Treated PC12 Cells

**Figure 1:**
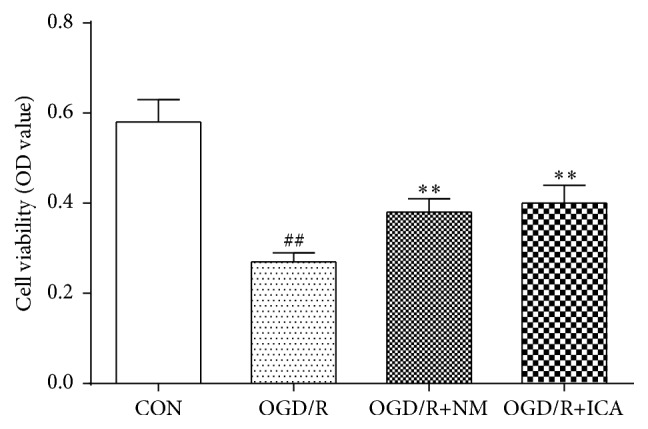
Effects of ICA on cell viability in OGD/R injured PC12 cells. OGD/R treated cells were subjected to 2 h OGD and 24 h reperfusion. ICA (10^−5^ M) or nimodipine (10 *μ*M) was given 1 h before OGD and acting through 2 h OGD. Normal control cells were refreshed with full culture medium and cultured under normoxic conditions. Cell viability was determined by MTT assay. Each group contained 10 samples. ^##^ *P* < 0.01 compared with normal control group. ^*∗∗*^ *P* < 0.01 compared with OGD/R group. CON: normal control, OGD/R+NM: OGD/R+ nimodipine.

Cell viability of OGD/R treated PC12 cells (0.27 ± 0.02) was markedly decreased compared with the normal cultured cells (0.58 ± 0.05, *P* < 0.01). However, cell viability of OGD/R treated cells was dramatically increased after ICA (10^−5^ M) or nimodipine (10 *μ*M) administration (0.40 ± 0.04 in ICA group, 0.38 ± 0.03 in nimodipine group, *P* < 0.01) (Figure 1). Our previous study showed that nimodipine could increase cell viability of OGD/R treated PC12 cells, so we set nimodipine as a positive control group [14].

### 3.2. ICA Increased Bcl-2 Expression and Decreased Bax and Cleaved Caspase-3 Expressions in OGD/R Treated PC12 Cells

**Figure 2:**
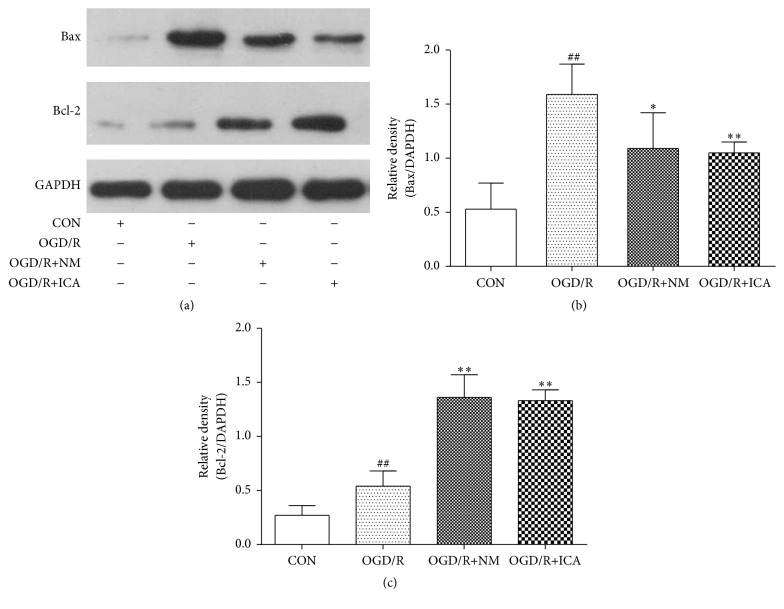
Effects of ICA on Bcl-2 and Bax expressions in OGD/R injured PC12 cells. OGD/R treated cells were subjected to 2 h OGD and 24 h reperfusion. ICA (10^−5^ M) or nimodipine (10 *μ*M) was given 1 h before OGD and acting through 2 h OGD. Normal control cells were refreshed with full culture medium and cultured under normoxic conditions. Bax ((a) and (b)) and Bcl-2 ((a) and (c)) protein expressions were measured by western blotting. Each group contained 6 samples. ^##^ *P* < 0.01 compared with normal control group. ^*∗∗*^ *P* < 0.01 compared with model control group. ^*∗*^ *P* < 0.05 compared with model control group. CON: normal control, OGD/R+NM: OGD/R+ nimodipine.

**Figure 3:**
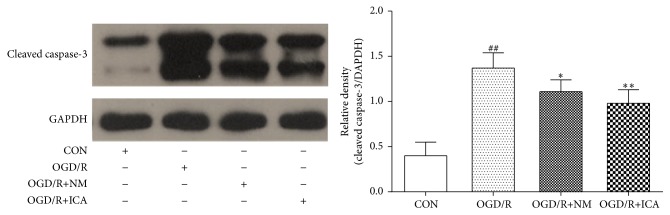
Effects of ICA on cleaved Caspase-3 expression in OGD/R injured PC12 cells. OGD/R treated cells were subjected to 2 h OGD and 24 h reperfusion. ICA (10^−5^ M) or nimodipine (10 *μ*M) was given 1 h before OGD and acting through 2 h OGD. Normal control cells were refreshed with full culture medium and cultured under normoxic conditions. Cleaved Caspase-3 expression was measured by western blotting. Each group contained 6 samples. ^##^ *P* < 0.01 compared with normal control group. ^*∗∗*^ *P* < 0.01 compared with model control group. ^*∗*^ *P* < 0.05 compared with model control group. CON: normal control, OGD/R+NM: OGD/R+ nimodipine.

Protein levels of Bcl-2, Bax, and cleaved Caspase-3 were significantly increased by OGD/R treatment (Bcl-2: 0.27 ± 0.12, *P* < 0.05; Bax: 0.82 ± 0.08, *P* < 0.01; cleaved Caspase-3: 1.37 ± 0.17, *P* < 0.01), compared to the normal control cells (Bcl-2: 0.13 ± 0.07; Bax: 0.34 ± 0.14; cleaved Caspase-3: 0.40 ± 0.20). However, ICA decreased Bax and cleaved Caspase-3 levels and increased Bcl-2 levels significantly in OGD/R treated cells (Bax: 0.61 ± 0.13, *P* < 0.01; cleaved Caspase-3: 0.98 ± 0.15, *P* < 0.01; Bcl-2: 0.53 ± 0.15, *P* < 0.01) (Figures 2 and 3). It was reported that nimodipine increased Bcl-2 expression and reduced expression of cleaved Caspase-3 and Bax in mice following middle cerebral artery occlusion (MCAO) injury, so we set nimodipine as a positive control group [16].

### 3.3. ICA Decreased Beclin-1 and LC3-II Expressions in OGD/R Treated PC12 Cells

**Figure 4:**
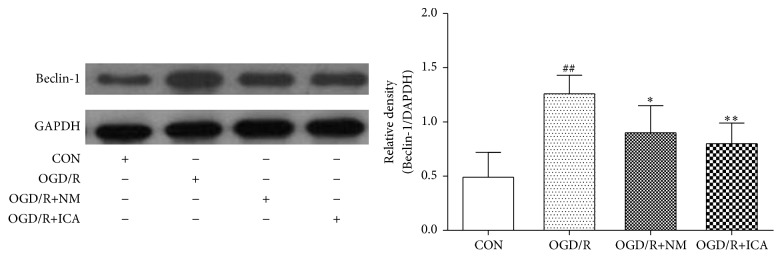
Effects of ICA on Beclin-1 expression in OGD/R injured PC12 cells. OGD/R treated cells were subjected to 2 h OGD and 24 h reperfusion. ICA (10^−5^ M) or nimodipine (10 *μ*M) was given 1 h before OGD and acting through 2 h OGD. Normal control cells were refreshed with full culture medium and cultured under normoxic conditions. Beclin-1 expression was measured by western blotting. Each group contained 6 samples. ^##^ *P* < 0.01 compared with normal control group. ^*∗∗*^ *P* < 0.01 compared with model control group. ^*∗*^ *P* < 0.05 compared with model control group. CON: normal control, OGD/R+NM: OGD/R+ nimodipine.

**Figure 5:**
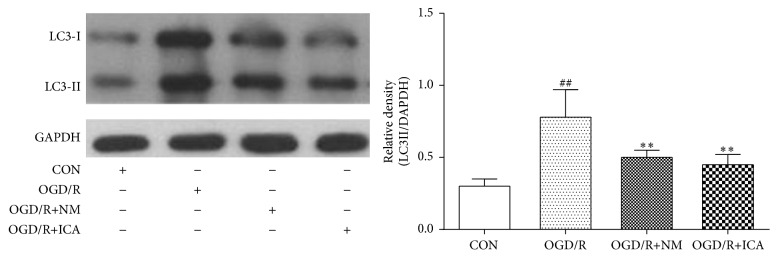
Effects of ICA on LC3II expression in OGD/R injured PC12 cells. OGD/R treated cells were subjected to 2 h OGD and 24 h reperfusion. ICA (10^−5^ M) or nimodipine (10 *μ*M) was given 1 h before OGD and acting through 2 h OGD. Normal control cells were refreshed with full culture medium and cultured under normoxic conditions. LC3II expression was measured by western blotting. Each group contained 6 samples. ^##^ *P* < 0.01 compared with normal control group. ^*∗∗*^ *P* < 0.01 compared with model control group. CON: normal control, OGD/R+NM: OGD/R+ nimodipine.

Protein levels of Beclin-1 and LC3-II were low in normal cultured cells (Beclin-1: 0.69 ± 0.06; LC3-II: 0.30 ± 0.05), but they were dramatically increased after OGD/R treatment (Beclin-1: 1.32 ± 0.17, *P* < 0.01; LC3-II: 1.15 ± 0.18, *P* < 0.01). However, ICA decreased Beclin-1 and LC3-II expressions significantly in OGD/R treated cells (Beclin-1: 0.92 ± 0.12, *P* < 0.01; LC3-II: 0.79 ± 0.18, *P* < 0.01) (Figures 4 and 5). Our previous study showed that nimodipine could reduce Beclin-1 expression and autophagosomes of OGD/R treated PC12 cells, so we set nimodipine as a positive control group [14].

## 4. Discussion

ICA possesses remarkable pharmacological activities on many kinds of central nervous system diseases. It can reduce neuronal injury caused by ischemic stroke in vitro and in vivo, attenuate learning and memory deficits, and alleviate depression [10, 11, 17]. Its potential mechanisms include anti-inflammation, antioxidation, and antiapoptosis [10–12]. Apoptosis is a process closely related to autophagy, and there are several cross talks between them. The role of ICA in autophagy and the cross talk between apoptosis and autophagy is obscure.

Apoptosis, also called programmed cell death, is regulated by a variety of apoptosis related proteins including Bcl-2 family proteins. Bcl-2 family proteins consist of antiapoptotic proteins such as Bcl-2 and Bcl-xL and proapoptotic proteins such as Bax and Bak. Bcl-2 and Bax levels are directly related to the occurrence of apoptosis. When Bcl-2 expression is increased, Bcl-2 binds to Bax to form heterodimers and inhibits the apoptosis. When Bax expression is increased, Bax forms homodimers and promotes apoptosis [7]. Caspase-3, the executor of apoptosis, can be activated by Bax homodimers [18]. When Caspase-3 is activated, procaspase-3 is cut into an active fragment cleaved Caspase-3 and then plays the role of proteolytic enzymes to promote apoptosis [19]. Therefore, cleaved Caspase-3 is a molecular level indicator which directly reflects cellular apoptosis [20]. In the present study, we showed that Bcl-2 protein expression was significantly decreased (as shown in Figure 2); Bax and cleaved Caspase-3 protein expression were dramatically increased in PC12 cells exposed to OGD/R, compared to normal control group, whereas these effects were significantly reversed by ICA treatment (as shown in Figures 2 and 3), suggesting that ICA inhibits apoptosis in PC12 cells through upregulation of Bcl-2 protein expression and downregulation of Bax protein expression.

Autophagy is a process of self-degradation through an autophagosomal-lysosomal pathway. It plays a key role in providing energy and raw material and turning over useless protein and organelles [1–3]. However, high level of autophagy may also aggravate cellular damage and result in autophagic cell death or apoptosis [4–6]. Protein light 1 chain microtubule-associated 3 (MAP1LC3) is a key protein that proves the occurrence of autophagy. LC3 converts to LC3-I and LC3-II during autophagy. LC3-II is a key component of autophagosome membranes [21], so LC3-II is a marker of autophagy. Beclin-1, an autophagy related protein, was first discovered in mammals. It can mediate other autophagic proteins attached to autophagosome membranes and reduce LC3-II accumulation [22]. Beclin-1 is also an important indicator to measure the degree of autophagy [23, 24]. As shown in Figures 4 and 5, ICA treatment reduced Beclin-1 and LC3-II protein expression in OGD/R treated PC12 cells. In our previous study, we demonstrated that Beclin-1 expression and autophagosomes were increased in PC12 cells subjected to 2 h of OGD, followed by 24 h of reperfusion, whereas the autophagy inhibitor 3-methyladenine (3-MA) reduced them in the OGD/R treated group [25]. The model was made the same as in this work. Since LC3-II is a component of autophagosome membranes, these results and our previous work suggest that ICA reduces autophagy. Bcl-2 is an important protein that inhibits apoptosis and autophagy. It not only combines with Bax to inhibit apoptosis but also binds to Beclin-1 to form a complex that inhibits Beclin-1 and autophagy activation, while maintaining resistance to apoptosis [7–9]. Our results, as shown in Figures 2 and 4, and these literatures suggest that ICA increases Bcl-2 protein expression, which further reduces the protein levels of Bax and Beclin-1.

Calcium overload leads to apoptosis and autophagy [26, 27]. Our previous studies have indicated that nimodipine, a calcium antagonist, can decrease intracellular free Ca^2+^ concentration and autophagy in PC12 cells subjected to OGD/R [27, 28]. Therefore, this work set nimodipine as a positive control group. This study demonstrated that ICA increased Bcl-2 protein expression, reduced the protein expression of Bax, Beclin-1, LC3-II, and cleaved Caspase-3, and enhanced cell viability in PC12 cells subjected to OGD/R. These results suggest that ICA may reduce the level of autophagy and apoptosis via activating Bcl-2-dependent cross talk between apoptosis and autophagy.

## References

[1] Alirezaei M., Kemball C. C., Flynn C. T., Wood M. R., Whitton J. L., Kiosses W. B. (2010). Short-term fasting induces profound neuronal autophagy. *Autophagy*.

[2] Wang P., Xu T.-Y., Wei K. (2014). ARRB1/*β*-arrestin-1 mediates neuroprotection through coordination of BECN1-dependent autophagy in cerebral ischemia. *Autophagy*.

[3] Ecker N., Mor A., Journo D., Abeliovich H. (2010). Induction of autophagic flux by amino acid deprivation is distinct from nitrogen starvation-induced macroautophagy. *Autophagy*.

[4] Siddiqui M. A., Mukherjee S., Manivannan P., Malathi K. (2015). RNase L cleavage products promote switch from autophagy to apoptosis by caspase-mediated cleavage of beclin-1. *International Journal of Molecular Sciences*.

[5] Cui C., Gao J., Cui Y. (2015). Chloroquine exerts neuroprotection following traumatic brain injury via suppression of inflammation and neuronal autophagic death. *Molecular Medicine Reports*.

[6] Uchiyama Y., Koike M., Shibata M. (2008). Autophagic neuron death in neonatal brain isehemia/hypoxia. *Autophagy*.

[7] Lalier L., Cartron P.-F., Juin P. (2007). Bax activation and mitochondrial insertion during apoptosis. *Apoptosis*.

[8] Salminen A., Kaarniranta K., Kauppinen A. (2013). Beclin 1 interactome controls the crosstalk between apoptosis, autophagy and inflammasome activation: impact on the aging process. *Ageing Research Reviews*.

[9] Ciechomska I. A., Goemans G. C., Skepper J. N., Tolkovsky A. M. (2009). Bcl-2 complexed with Beclin-1 maintains full anti-apoptotic function. *Oncogene*.

[10] Liu B., Xu C., Wu X. (2015). Icariin exerts an antidepressant effect in an unpredictable chronic mild stress model of depression in rats and is associated with the regulation of hippocampal neuroinflammation. *Neuroscience*.

[11] Li L., Zhou Q.-X., Shi J.-S. (2005). Protective effects of icariin on neurons injured by cerebral ischemia/reperfusion. *Chinese Medical Journal*.

[12] Li F., Gao B., Dong H., Shi J., Fang D. (2015). Icariin induces synoviolin expression through NFE2L1 to protect neurons from ER stress-induced apoptosis. *PLoS ONE*.

[13] Wang L., Zhang L., Chen Z.-B., Wu J.-Y., Zhang X., Xu Y. (2009). Icariin enhances neuronal survival after oxygen and glucose deprivation by increasing SIRT1. *European Journal of Pharmacology*.

[14] Mo Z.-T., Fang Y.-Q., He Y.-P., Zhang S. (2012). *β*-Asarone protects PC12 cells against OGD/R-induced injury via attenuating Beclin-1-dependent autophagy. *Acta Pharmacologica Sinica*.

[15] Zeng K.-W., Liao L.-X., Zhao M.-B. (2015). Protosappanin B protects PC12 cells against oxygen-glucose deprivation-induced neuronal death by maintaining mitochondrial homeostasis via induction of ubiquitin-dependent p53 protein degradation. *European Journal of Pharmacology*.

[16] Zhao P., Zhou R., Zhu X.-Y. (2015). Matrine attenuates focal cerebral ischemic injury by improving antioxidant activity and inhibiting apoptosis in mice. *International Journal of Molecular Medicine*.

[17] Jin F., Gong Q.-H., Xu Y.-S. (2014). Icariin, a phoshphodiesterase-5 inhibitor, improves learning and memory in APP/PS1 transgenic mice by stimulation of NO/cGMP signalling. *International Journal of Neuropsychopharmacology*.

[18] Cregan S. P., MacLaurin J. G., Craig C. G. (1999). Bax-dependent caspase-3 activation is a key determinant in p53-induced apoptosis in neurons. *The Journal of Neuroscience*.

[19] Lazebnik Y. A., Kaufmann S. H., Desnoyers S., Poirier G. G., Earnshaw W. C. (1994). Cleavage of poly(ADP-ribose) polymerase by a proteinase with properties like ICE. *Nature*.

[20] Chakravarti A., Zhai G., Suzuki Y. (2004). The prognostic significance of phosphatidylinositol 3-kinase pathway activation in human gliomas. *Journal of Clinical Oncology*.

[21] Tanida I., Ueno T., Kominami E. (2004). LC3 conjugation system in mammalian autophagy. *International Journal of Biochemistry and Cell Biology*.

[22] Su X., Wang X., Liu Q., Wang P., Xu C., Leung A. W. (2015). The role of Beclin 1 in SDT-induced apoptosis and autophagy in human leukemia cells. *International Journal of Radiation Biology*.

[23] Backer J. M. (2008). The regulation and function of Class III PI3Ks: novel roles for Vps34. *Biochemical Journal*.

[24] Liang X. H., Jackson S., Seaman M. (1999). Induction of autophagy and inhibition of tumorigenesis by beclin 1. *Nature*.

[25] He Y., Mo Z., Xue Z., Fang Y. (2013). Establish a flow cytometric method for quantitative detection of Beclin-1 expression. *Cytotechnology*.

[26] He L., Poblenz A. T., Medrano C. J., Fox D. A. (2000). Lead and calcium produce rod photoreceptor cell apoptosis by opening the mitochondrial permeability transition pore. *The Journal of Biological Chemistry*.

[27] Vicencio J. M., Lavandero S., Szabadkai G. (2010). Ca^2+^, autophagy and protein degradation: thrown off balance in neurodegenerative disease. *Cell Calcium*.

[28] Mo Z. T., Fang Y. Q., He Y. P., Ke X. H. (2012). Change of Beclin-1 dependent on ATP, [Ca^2+^]_i_ and MMP in PC12 cells following oxygen-glucose deprivationreoxygenation injury. *Cell Biology International*.

